# Supplementary material for: How high energy fluxes may affect Rayleigh–Taylor instability growth in young supernova remnants
Source: Nat Commun. 2018 Apr 19;9:1564. doi: 10.1038/s41467-018-03548-7 (PMC5908785; doi:10.1038/s41467-018-03548-7)
Supplement: Supplementary file 1 — Supplementary Information [file 41467_2018_3548_MOESM1_ESM.pdf]

Supplementary Note 1: Experimental Details

The experimental target (see Supplementary Figure 1) is attached to the gold hohlraum, and consists of a dense layer followed by a lower density layer to create a well-known density gradient. The dense plastic includes an initial layer of CH, in which the soft x-rays from the hohlraum create the pressure impulse, a thin gold layer (to attenuate the gold M-band emissions from the laser spots), and a layer of plastic within which the blast wave forms. The plastic layer is structured so that it includes a 400-μm-wide tracer strip of iodinated plastic that is transverse to the diagnostic line of sight, sandwiched between two layers of polyimide that are of the same density. The outer surface of the plastic was machined to produce the modulations of 120-μm wavelength and 6-μm amplitude that initiate the unstable behavior. Beyond this surface is the SiO_2 foam at 0.02 g cm^{-3}.

The experiments discussed in this manuscript were performed at the National Ignition Facility[1] in April and June, 2015. Identical targets were fielded in a sequence of realizations of the experiment under two conditions. High-flux and low-flux experiments used targets with nominally identical target components (see Supplementary Figure 1), but differed in the laser pulses, as described below, and in the external shielding that was necessary to reduce the x-ray background generated in the high-flux case. We used a sequence of laser shots to observe the evolution of structure at an interface for each of the high-flux and low-flux cases.

Supplementary Figure 2a shows the profiles of laser power delivered to the targets, by 64 laser beams.

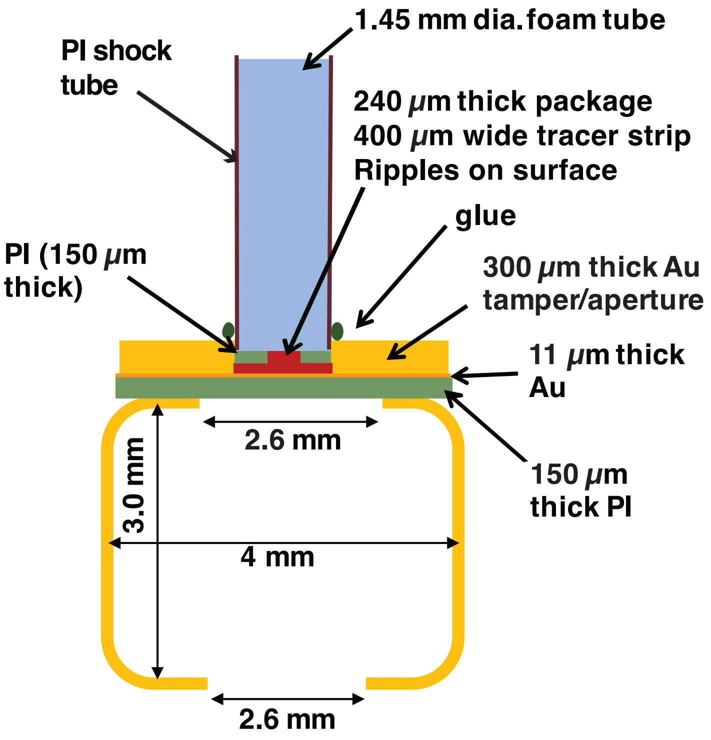

Supplementary Figure 1: **Target Schematic** A cross section of the essential elements in the target for the National Ignition Facility experiments. The polyimide layer is denoted PI in the figure. The hohlraum and most components are cylindrically symmetric, but the tracer strip extends only in the direction perpendicular to the diagnostic line of sight.

In the high-flux case, all beams had the same temporal profile of power, with a peak power of ~ 150 TW at the end of the 3 ns pulse. The low-flux pulse was divided into two portions, to reduce the potential for damage to the laser. These were staggered in time, so the cumulative pulse duration was ~ 6 ns with a peak power near 36 TW. Supplementary Figure 2b shows the hohlraum temperatures measured by the Dante diagnostic.[2] Hohlraum temperatures in similar ranges (240 - 340 eV) have been studied and calibrated by previous platforms.[3;4;5;6] The high-flux hohlraum temperature T_{R} was measured to be approximately 40% greater than the low-flux temperature, consistent with the expected scaling of temperature with laser power as $T_{\mathrm{R}} \propto P^{0.286}$. Twenty-eight other beams provided energy for x-ray radiography.

Supplementary Note 2: Simulation Details and Analysis

CRASH simulations aided in the analysis of this work. We were able to ascertain several parameters that we could not directly measure in the experiment. This includes the Atwood number and interface acceleration, both of which are components of the Rayleigh-Taylor growth factor, τ. The interface acceleration, $g(t)$, is the time derivative of the velocity of the interface, which is determined from a simulation without a seeded perturbation (*i.e.*, flat plastic-foam interface). For the high-flux case $g(t) = -18.8t^{-0.8}$ and in the low-flux case $g(t) = -11.5t^{-0.9}$. In both cases, g is in μm ns^{-2} and t is in ns. For the Atwood number the material densities are taken at the local maximum density and local minimum density near the interface position ignoring features that are caused by material property discontinuities. In both cases, the post-shock Atwood number is taken relatively constant and is 0.5 and 0.7 in the high-drive and low-drive case, respectively. Other quantities are extracted from the simulation for comparing to the experiment. This includes the position of the spike and bubble. Several methods were used, which account for the error bars in 3 of the main text.

As an independent way to examine the effect of radiative energy fluxes in the lab experiment, we did simulations for the high-flux conditions in which we caused the radiation from the shocked foam to be small by reducing the opacity by a factor of 1000, so that the shocked-foam layer became optically thin. Supplementary Figure 3 shows the results. These simulations used periodic upper and lower boundary conditions, and modeled one-half wavelength in an x-y geometry.

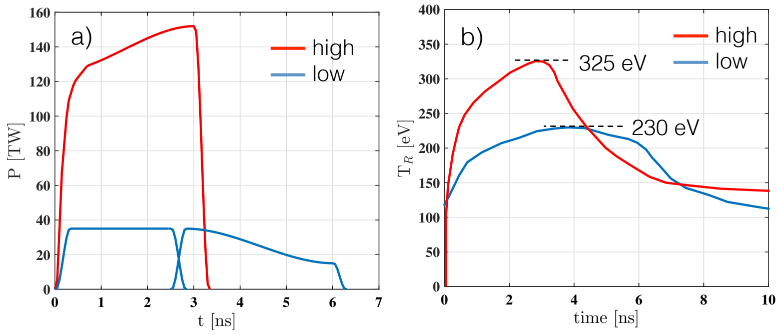

Supplementary Figure 2: **Laser pulse and temperature profiles for experiment** a) The laser pulses used in the high-flux and low-flux experiments. The total energy delivered by the laser varied by ±2% over the high-flux shots and ±1% over the low-flux shots. The low-flux pulse was split into two portions to reduce the risk of damage to the laser. b) The measured soft-x-ray temperature for each case.

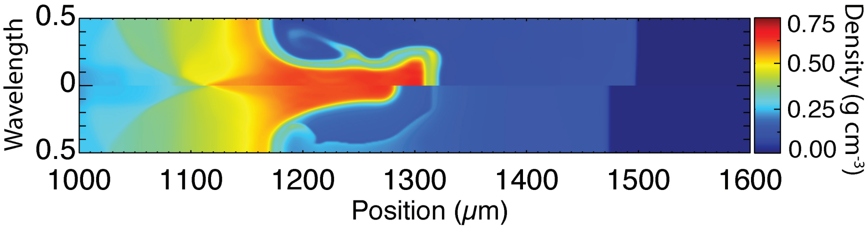

Supplementary Figure 3: **Comparison of simulated nominal and reduced opacities** Simulation results of density using the high flux from the hohlraum onto the dense plastic at 13 ns. The lower panel uses the nominal opacity for the SiO_2 foam. The upper panel reduces this opacity by a factor of 1,000, which has the implication that the radiation flux produced by the shocked foam is greatly reduced. One can see that the effect of having the nominal radiative energy flux from the foam to the interface is that the spike-to-bubble distance is reduced and the "mushroom-cap" structure at the spike tip is eliminated.

Supplementary Note 3: Hydrodynamics, Instabilities, and Energy Fluxes

During the explosion of a supernova (SN), a blast wave sweeps through the star, creating flowing, stellar ejecta. (A blast wave is composed of a shock front followed immediately by a density and pressure decrease.) A key event that occurs as the blast wave approaches the surface of the star happens when the region ahead of the blast wave that is heated by radiation from it reaches the stellar surface so energy can escape. The escape of radiation leads to formation of a dense shell of stellar ejecta.[8;9] The dense shell acts like a piston to drive a forward shock into the circumstellar medium (CSM).[10] Once the radiation can escape ahead of the shock, the radiation pressure remains negligible, even though the shocked matter may become optically thick. As the forward shock sweeps up mass and the pressure in the ejecta decreases through expansion, the structure decelerates and a reverse shock forms in the ejecta. There is an interface, also known as the contact surface, with an abrupt drop in density, between the two shocks. The interface forms when the SN shock reaches the change in density profile at the stellar surface and proceeds to drive a shock into the CSM, initiating the formation of the supernova remnant (SNR). The interface between the shocked ejecta and the shocked CSM thus arises hydrodynamically, and the transition across it will initially occur in a few ion-ion mean-free-paths. Supplementary Figure 4 shows the primary elements of the structure just described. Because pressure is continuous across such an interface, the temperature is much higher in the shocked, less dense CSM than in the denser ejecta. This leads to the possibility of radiative or conductive transport of

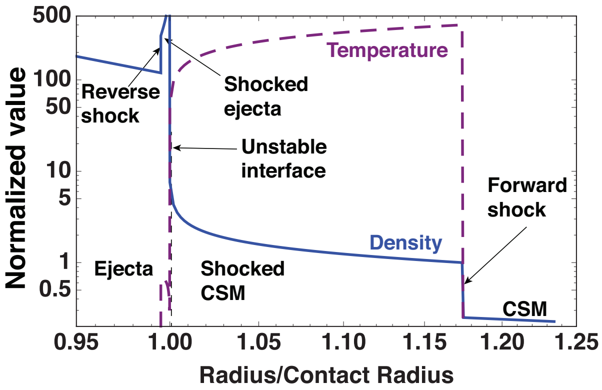

Supplementary Figure 4: **Self-similar profiles of relative density and temperature** Profiles from the self-similar formulation of Chevalier, where $n = 30$ and $s = 1.7$, which includes hydrodynamic effects only[7]

energy into the denser ejecta, which in turn can affect the evolution of the Rayleigh-Taylor (RT) instability at the interface by ablating material from it. In addition, there is a phase when radiation from matter heated by the reverse shock also might affect the RT.

Let us consider the structure of SNRs from Type II SNe, based on the specific models of SN1993J in Suzuki and Nomoto[11] and informed by the discussion in Fransson et al.[12] These authors evaluated many aspects of the data, from the radio to the x-ray, and developed reference profiles of the parameters that were consistent with these data. Other earlier work[13;7] discusses more basic descriptions of SNR development for a range of ejecta and CSM profiles, implying similar consequences. They discuss how the parameters vary in response to assumptions about stellar mass loss rate, wind speed, and reference ejecta density, but the results scale very weakly with variations of these parameters.

The ejecta-density profile is[7]

$$\rho_{\rm ej} = \rho_{\rm o} \left(\frac{t}{t_{\rm o}}\right)^{(n-3)} \left(\frac{r}{r_{\rm o}}\right)^{-n}. \tag{1}$$

By fitting several aspects of the data from SN1993J, Suzuki et al.[11] find reference density $\rho_{\rm o} = 4.5 \times 10^{-14}$ g cm^{-3} at radius $r_{\rm o} = 5 \times 10^{13}$ cm and $n \sim 30$. They also find a CSM-density profile of $\rho_{\rm CSM} = \rho_{\rm o}(r_{\rm o}/r)^s$, with $s = 1.7$. We note that Fransson et al[14] prefer $s = 2$. We use the model of Suzuki et al.[11] below, but note that the conclusions drawn are not sensitive to the exact choice of parameters, and also would apply to the cases having much shallower ejecta profiles that are thought to be more typical. The unshocked ejecta are homologous so $v = r/t$ and $v_{\rm o} = r_{\rm o}/t_{\rm o}$, with $v_{\rm o} = 2.5 \times 10^9$ cm s^{-1} so $t_{\rm o} = 2 \times 10^4$ s. In the model of SN1993J, the self-similar structure forms after the shock front moves into the CSM, which has a much shallower density profile than the ejecta do. A density difference of a few hundred occurs at the interface, for $n = 30$ and $s = 1.7$. Supplementary Figure 4 shows the corresponding density profile. Note that the density jump at the interface is about a factor of 250. The pressure, in contrast, is (as required by fundamental fluid dynamics) continuous across the interface. If the dense shell mentioned above is even denser than the shocked ejecta considered here, then there will be a denser, cooler shell inserted at the interface location. The interface position is given by Chevalier et al.[7] as

$$r_{\rm c} = r_{\rm o}(t/t_{\rm o})^{(n-3)/(n-s)}. \tag{2}$$

In consequence, the interface for this case decelerates at ~ 25 cm s^{-2} at 0.1 years. As a result, the interface is RT-unstable. The reverse-shock and forward-shock positions are fixed multiples of $r_{\rm c}$, $\chi_{\rm r}$ and $\chi_{\rm f}$, respectively. One must find these numerically. For $n = 30$ and $s = 1.7$, one has $\chi_{\rm r} = 0.995064$ and $\chi_{\rm f} = 1.174$. One thus has the speed of the reverse shock, in the frame of the star, is

$$v_{\rm rs} = \chi_{\rm r} v_{\rm o} \frac{(n-3)}{(n-s)} \left(\frac{t}{t_{\rm o}}\right)^{(s-3)/(n-s)}, \tag{3}$$

while the speed of the flow entering the shock in the same frame is

$$v_{\rm in} = \frac{\chi_{\rm r} r_{\rm c}}{t} = \chi_{\rm r} v_{\rm o} \left(\frac{t}{t_{\rm o}}\right)^{(s-3)/(n-s)}. \tag{4}$$

These imply that the speed of the flow entering the reverse shock, in the reverse-shock frame of reference, is

$$v_{\rm rso} = v_{\rm in} - v_{\rm rs} = \chi_{\rm r} v_{\rm o} \frac{(3-s)}{(n-s)} \left(\frac{t}{t_{\rm o}}\right)^{(s-3)/(n-s)}. \tag{5}$$

The mass density of the ejecta at the reverse shock is

$$\rho_{\rm rs} = \rho_{\rm o} \left(\frac{t}{t_{\rm o}}\right)^{(n-3)} \left(\frac{\chi_{\rm r} r_{\rm c}}{r_{\rm o}}\right)^{-n} = \frac{\rho_{\rm o}}{\chi_{\rm r}^n} \left(\frac{t}{t_{\rm o}}\right)^{-s(n-3)/(n-s)}. \tag{6}$$

The mechanical-energy flux driving the shock-heating of the shocked matter by the reverse shock is thus of order

$$F_{\rm mech} = \rho_{\rm ej} v_{\rm rso}^3 = \frac{\rho_{\rm o} v_{\rm o}^3}{\chi_{\rm r}^{(n-3)}} \left(\frac{3-s}{n-s}\right)^3 \left(\frac{t}{t_{\rm o}}\right)^{-(ns+9-6s)/(n-s)} \tag{7}$$

$$= 9.65 \times 10^{-5} \rho_{\rm o} v_{\rm o}^3 \left(\frac{t}{t_{\rm o}}\right)^{-1.76} = \frac{1.84 \times 10^5}{t_{\rm yr^{1.76}}} {\rm ergs\ cm^{-2} s^{-1}},$$

in which the second line is for the reference parameters given above.

The forward shock speed is

$$v_{\rm fs} = \chi_{\rm f} v_{\rm o} \frac{(n-3)}{(n-s)} \left(\frac{t}{t_{\rm o}}\right)^{(s-3)/(n-s)} = \frac{2.4 \times 10^9}{t_{\rm yr}{}^{0.046}} {\rm cm\ s^{-1}}, \tag{8}$$

in which the final term is for the model of SN1993J. For a hydrogen CSM cool enough that the shock is strong, as is likely, and polytropic index $\gamma = 5/3$, the resulting post-shock temperature, assuming no electron-ion equilibration, is

$$T_{\rm i,cs} = 2.27 \times 10^9 \mu_{\rm s} \frac{(n-3)^2}{(n-s)^2} V_4^2 \ {\rm K} \tag{9}$$

where $\mu_{\rm s}$ is the mean mass per particle in amu and V_4^2 is the shocked ejecta velocity in 10^4 km s^{-1}. For SN1993J $\mu_{\rm s}$ is ~ 1 according to Baron et al.[15] and the velocity is defined above so that

$$T_{\rm i,cs} = \frac{7.8 \times 10^5}{t_{\rm yr}^{0.092}} \ {\rm eV} = \frac{9.1 \times 10^9}{t_{\rm yr}^{0.092}} \ {\rm K}. \tag{10}$$

Electron heating is fairly slow. The calculations[12] suggest that the electron temperature in the shocked CSM near the interface is about 10% of the value from Supplementary Equation 10. We take this temperature to be $T_{\rm e,cs} = 0.1 T_{\rm i,cs}$. We do not consider here potential variations over time in the degree of equilibration. Pressure is proportional to temperature in the regime of interest and is also continuous across the interface for pure hydrodynamics. The corresponding temperature in the shocked ejecta is smaller than that in the shocked CSM by the same ratio as the density increases. For the 250-fold density jump, the temperature in the shocked ejecta, if the material is the same, would thus be $\sim 3 \times 10^3 t_{\rm yr}^{-0.092}$ eV or $\sim 4 \times 10^7 t_{\rm yr}^{-0.092}$ K. This could be reduced by electron-ion equilibration.

However, pure hydrodynamics may well be insufficient to accurately predict the structure of the young SNR. As the shock structure at the interface between the CSM and the dense ejecta forms, heat flow is possible by radiation and by electron heat conduction. There have been discussions of heat conduction for the SN case in 1-dimension, although the problem has not been solved[16;17]. We are not aware of any multi-dimensional simulations including these effects. Standard models indicate that the shocked CSM is probably never optically thick, and probably never cools strongly by radiative losses. In contrast, the radiative losses from the shocked layer produced by the reverse shock are found to be large enough to cool it significantly. The energy flux associated with this cooling, for a layer of thickness D, is

$$F_{\rm rad} = n_{\rm e} n_{\rm i} \Lambda D \sim \frac{3.6 \times 10^7}{t_{\rm yr}^{2.26}} \ {\rm ergs\ cm^{-2} s^{-1}}, \tag{11}$$

in which $n_{\rm i}$ is the ion density, Λ is the cooling function, and the final term is based on the model of SN1993J just described. Nymark et al.[18] give a fit for Λ (we used the one for a solar composition here). Their estimate of the rate of expansion of this layer caused by the hydrodynamic expansion of the SNR with the rate of contraction caused by radiative cooling indicate that the layer is likely to collapse for some period of time, of a duration determined by the details of their calculation. This is also indicated by the fact that $F_{\rm rad} >> F_{\rm mech}$ for a period of time, showing that the hydrodynamic model is not sufficient to accurately

describe the behavior. The radiative cooling carries energy into the collapsed ejecta (and whatever remains of the the dense shell produced by the shock breakout dynamics, mentioned above), but the radiation is not able to penetrate the dense shell until much later. At that late time, the heating of the dense shell might lead to an expansion of its outer surface (the interface) and potentially to stabilization of RT. This was our original hypothesis regarding potential effects of energy fluxes in the SNR. However, we discovered that heat conduction is an earlier and more persistent source of ablation at the interface.

As just described, radiative cooling of the shocked ejecta tends to increase the density and decrease the temperature of the shocked ejecta. However, heat conduction from the shocked CSM will resist this (as would magnetic field accumulation, if it were sufficient). In many contexts, heat conduction is a diffusive process. However this requires that the temperature scale length is quite large (>> 30 mean-free-paths for the high-temperature particles[19]). In contrast, in the regime of interest here, the collisional mean-free-path is larger than the temperature scale lengths seen in the referenced calculations. This is a common regime for laser-produced plasmas and other strongly-heated laboratory plasmas. In such cases, the electron heat flux $Q_{\rm e}$ can be approximated as a fraction, of order 10%, of the free-streaming heat flux, so that

$$Q_{\rm e} = 0.1 n_{\rm e} m_{\rm e} v_{\rm e,cs}^3 \text{ ergs cm}^{-2}\text{s}^{-1}, \tag{12}$$

in which the electron density is $n_{\rm e} = \rho_{\rm cs}/m_{\rm p}$ for H, the electron mass is $m_{\rm e}$, and the electron thermal velocity in the shocked CSM is $v_{\rm e,cs} = \sqrt{k_{\rm B} T_{\rm e,cs}/m_{\rm e}}$, with Boltzmann constant $k_{\rm B}$. The collisional electron mean free path within the CSM is of order the layer thickness, so this should be a reasonable model if the magnetic field is small enough. The density in the shocked CSM at the interface is about 10 times the density in the unshocked CSM, $\rho_{\rm f,cs}$,so one has at early times for a hydrogen CSM

$$Q_{\rm e,cs} = \frac{\rho_{\rm f,cs} m_{\rm e}}{m_{\rm p}} \left(\frac{k_{\rm B} T_{\rm e,cs}}{m_{\rm e}}\right)^{3/2} = \frac{2.0 \times 10^8}{t_{\rm yr}^{1.76}} \text{ ergs cm}^{-2}\text{s}^{-1}, \tag{13}$$

in which the proton mass is $m_{\rm p}$. One can see that, in this model, the incoming energy flux by heat conduction is larger than the incoming mechanical energy flux, by a factor of 1,000 at all times. The point here is that this is large enough that this energy flux may be a dominant effect in establishing the structure of the layer. This may be true even if the energy flux is reduced, either because of magnetization or as it moderates the density and temperature gradients found in the purely hydrodynamic model.

As it turns out the model just discussed is also relevant to the magnetized, turbulent state that is inferred to exist in SN1993J. From an analysis of the measured radio spectra, attributed to synchrotron emission[14] infer that the magnetic field in the shocked CSM is probably tens of Gauss during the time period when energy-flux effects might be significant. This represents a significant fraction (their number is 14%) of the local thermal energy. For this high a magnetic field, the electrons would be very well magnetized. The condition for the electrons to be well-magnetized is $\omega_{\rm ce}\nu_{\rm ee} = 1$, in which $\omega_{\rm ce} = eB/m_{\rm e}c$, for Gaussian cgs units with electric charge e, magnetic field B, and light speed c, and $\nu_{\rm ee} = 4.2 \times 10^{-6} n_{\rm e} \log\Lambda/T_{\rm eV}^{3/2}$, with electron temperature in eV being $T_{\rm eV}$ and Coulomb logarithm being $\log\Lambda$. One finds the magnetic field required for magnetization to matter to be 100 μGauss $\sim$ 10 nT in the SNR and $\sim$ 100 MGauss in the lab experiment. The field is far smaller than 100 MGauss in the lab, but far larger than 100 μGauss in the SNR.

It turns out that, in such a magnetized case, the heat transport is also reasonably estimated by Supplementary Equation 12. Ryutov et al.[20] discuss heat transport in a generic, magnetized plasma. Assuming the magnetic field within the CSM to be turbulent, as seems likely and is the case in the Solar wind, they find a minimum value for the kinematic coefficient of heat conduction to be $r_{\rm Li} v_{\rm e,cs}$, in which the ion Larmor radius of gyration in the magnetic field is $r_{\rm Li}$. If the scale height of the temperature change is $10 r_{\rm Li}$, then this implies a heat flux of order that of Supplementary Equation 12. If the temperature gradient decreases, the heat flux would go down, but by then the surface of the dense ejecta will be heated and will ablate, influencing the RT instability. A simpler way to make the same point is that in the turbulent- (Bohm-) diffusion limit, the electrons leave the hotter matter by moving some fraction ($\sim 1/16$) of a gyro orbit radius in a time ($1/\omega_{\rm ci}$), where $\omega_{\rm ci}$ is the electron cyclotron frequency in radians s^{-1}. The ratio of this distance to this time gives a speed $\sim v_{\rm e,cs}/16$ and thus a heat flux roughly consistent with Supplementary Equation 12.

As the hot electrons from the shocked CSM penetrate the dense matter, the equilibration rate for heating the cool electrons is much faster than that for heating the ions. An order-of-magnitude model that works well for laser-heated plasmas is to assume the cool electrons are heated until they carry the incoming heat flux. The implied temperature in the ejecta is then

$$T_{\mathrm{ej}} = \left(\frac{n_{\mathrm{cs}}}{n_{\mathrm{ej}}}\right)^{2/3} T_{\mathrm{e,cs}}, \tag{14}$$

in which the electron densities in the shocked CSM and shocked ejecta are n_{cs} and n_{ej}, respectively. For $n_{\mathrm{ej}}/n_{\mathrm{cs}} = 250$ at the interface, this gives a value of T_{ej} that is about 6 times larger than one finds at equal pressure. The heat front penetrates the ejecta at near the electron thermal velocity, which is $\sim 3 \times 10^4$ km s^{-1}.

What is significant here is that the heat flow will produce an increase of pressure in the dense layer and drive material away from the interface, much like a rarefaction does. The sound speed is the speed at which matter from the dense layer flows through the interface in a centered rarefaction wave, and should reasonably characterize the present case. The sound speed at T_{ej} just given, for a H plasma, is $600/t_{\mathrm{yr}}^{0.046}$ km s^{-1}, and so this should reasonably characterize the speed of removal of the dense material from the interface.

The basic physics produced by energy transport to material on the dense side of an interface is known as ablative stabilization of RT. RT involves the amplification of a wave the propagates along the interfacial surface; the removal of surface material has a stabilizing effect. This process is very important for inertial confinement fusion. Betti et al.[21] and Bose et al.[22] find a modified rate of exponentiation for RT at an interface to be

$$\gamma_{\mathrm{RT}} = \alpha\sqrt{\frac{kg}{1+kL}} - \beta k v_{\mathrm{a}}, \tag{15}$$

in which the wavenumber of the surface wave is k, the deceleration of the interface is g, and the speed at which material is ablated from the dense matter is v_{a}. The density scale height across the interface is L, and we will take kL to be small. The two parameters α and β depend on various details, are of order unity, and will be taken to be 1 here. The ablation of material thus completely stabilizes RT for wavenumbers above g/v_{a}^2, so RT is unstable for wavelengths longer than $2\pi v_{\mathrm{a}}^2/g$.

Note that the ablation quantified by v_{a} can occur in consequence of any energy flux reaching the interface. In the laboratory context, there is one study of the impact of heat conduction on RT. Modica et al.[23] found that heat conduction could affect the details of the structures produced in the nonlinear phase of RT. They studied a system morphologically similar to the experiments described here, but having smaller normalized energy fluxes than those produced in the high-flux experiment reported in the present paper.

We can use the estimate of v_{a} as the sound speed of the heated ejecta, and the value of the deceleration given above, to obtain the minimum unstable wavelength for RT, as

$$\lambda_{\mathrm{min}} = 9.3 \times 10^{15} t_{\mathrm{yr}}^{0.954}, \tag{16}$$

so that

$$\frac{\lambda_{\mathrm{min}}}{r_{\mathrm{c}}} = 0.166, \tag{17}$$

where r_{c} is the interface position defined in Supplementary Equation 2. Supplementary Equation 17 corresponds to a mode number of ~ 40. The fastest-growing mode will be of lower mode number. Purely hydrodynamic simulations of supernova remnants (SNRs), for example by Chevalier et al.[24] typically show RT producing large-scale structures having $\lambda \sim 0.1R_{\mathrm{s}}$ and substantial smaller-scale structure. (The radius of the shock is R_{s}.) The analysis here suggests that only the longest-wavelength features may be present. The stabilization of shorter-wavelength modes may prevent the development of narrow spikes and of a more turbulent unstable layer. There is some suggestive evidence for such an effect in the case of SNR E0102.2-72, shown in the main text.

The heat flux might be reduced in two ways, reducing the stabilizing effect. A two-stream instability may develop near the interface, when the bulk electrons flow toward the hotter matter to neutralize the current carried inward by the energetic electrons. The consequent ion acoustic turbulence reduces the inward heat flux, but at the cost of more intense local heating. In the present case, this might turn out to increase or to reduce the ablation, depending upon where the acoustic turbulence was most intense. Alternatively, if the magnetic field were sufficiently strong and coherent, and in a direction transverse to that of the shock, this could reduce the inward electron heat flux.

Supplementary Note 4: Dimensionless parameters

To construct dimensionless parameters for the systems of interest, we first need to specify the dimensional parameters involved. Here SEL refers to the shocked ejecta layer while SCSM refers to the layer of shocked CSM. Supplementary Table 1 shows the parameters for the experiment, based where necessary on simulations that reasonably match the data, and for the SNR, based on the discussion above. The relevant length for the physics of interest here is the distance between the shocks, L, and take the velocity relevant to the Reynolds number, U, to be the rate at which they separate.

Scale Parameter	SN1993J	NIF experiment
Intershock distance L (cm)	$2.8 \times 10^{14} t_{\rm yr}^{0.95}$	0.02
Shock separation speed U (cm^{-1})	$3.0 \times 10^{8} t_{\rm yr}^{-0.046}$	6.8×10^{6}
Ejecta density at RS (g cm^{-3})	$3.4 \times 10^{-19} t_{\rm yr}^{-1.6}$	0.026
SEL Density (g cm^{-3})	$1.4 \times 10^{-16} t_{\rm yr}^{-1.6}$	0.5
SCSM Density (g cm^{-3})	$9 \times 10^{-19} t_{\rm yr}^{-1.6}$	0.18
SEL Temperature (eV)	$3800 t_{yr}^{-0.092}$	20
SCSM Temperature (eV)	$7.8 \times 10^{5} t_{\rm yr}^{-0.092}$	80
RS Velocity (km s^{-1})	$1.7 \times 10^{4} t_{\rm yr}^{-0.046}$	35
FS Velocity (km s^{-1})	$2 \times 10^{4} t_{\rm yr}^{-0.046}$	170
Z	1	2
A	1	20
$F_{\rm mech}$ (ergs cm^{-2}s^{-1})	$1.8 \times 10^{5} t_{\rm yr}^{-1.76}$	2.8×10^{19}
$F_{\rm rad}$ (ergs cm^{-2}s^{-1})	$3.6 \times 10^{7} t_{\rm yr}^{-2.26}$	4.2×10^{19}
$Q_{\rm e}$ (ergs cm^{-2}s^{-1})	$2.0 \times 10^{8} t_{\rm yr}^{-1.76}$	1.7×10^{19}

Supplementary Table 1: **Parameters used to calculate the values in Supplementary Table 2**. RS Velocity is the speed of the matter entering the reverse shock in its rest frame. Laboratory values are taken at 13 ns from radiation hydrodynamics simulations results for the experiment performed in a high-radiative-flux regime and the astrophysical values are based on the model of SN1993J in Suzuki et al. [11].

We use the parameters in Supplementary Table 1 to evaluate dimensionless parameters of interest, shown in Supplementary Table 2. Whether the plasma behaves as a fluid is determined by the ion-ion collisionality, and so we have evaluated the collisional mean-free path in the main text as the mean free path for ion-ion collisions is $\lambda_{\rm c} = v_{\rm i}/\nu_{\rm ii}$, in which $v_{\rm i}$ is the ion thermal velocity and $\nu_{\rm ii}$ is the ion-ion collision frequency where

$$\nu_{\rm ii} = 4.80 \times 10^{-8} Z^4 n_{\rm i} \ln\Lambda {\rm T}_{\rm i}^{-3/2} {\rm sec}^{-1} \tag{18}$$

and we assume that $T_{\rm i} = 10 T_{\rm e}$. The electrons are nearly collisionless in the shocked CSM, with implications we just discussed, but are collisional in the cooler, denser shocked ejecta. And the ions, with their shorter

mean-free path, are collisional in both regions. We evaluate the Reynolds number as $Re = UL/\nu_{\rm i}$, where the ion viscosity is $\nu_{\rm i}$ is the collisional value from Ryutov et al.[20], evaluated for the shocked ejecta. A finite and plausible magnetic field is required for the shocked CSM to behave as a fluid. For the Peclet number in the diffusive regime, we have LU/χ, where χ is the thermal diffusivity. However, for free-streaming heat flow the heat flux is $Q = \frac{3}{2} f p v_{\rm the}$ with electron thermal speed $v_{\rm the}$ and flux-limiter f, which is about 0.1. Therefore, in the free-streaming regime, we have $Pe_{\rm free} = 2U/3fv_{\rm the}$.

To further consider the similarity of the systems we look at the Ryutov number, $Ry = v^*(\rho^*/P^*)^{1/2}$ (sometimes called the Euler number). When comparing two systems, most dimensionless numbers do not need to be the same, simply in the same regime. However, for two systems to fulfill the conditions for hydrodynamics similarity, as detailed by Ruytov et al.[20], Ry must be the same in both systems. While Ry for both systems is similar (see Supplementary Table 2), these estimates are based on many assumptions and could vary by at least an order of magnitude. In general, scaling to a specific object is not the main goal of this work, which is rather to show the importance of energy fluxes in the evolution of young supernova remnants.

To characterize the impact of the heat flow into the layer inside the interface, we compare it with the mechanical energy injected by the reverse shock. We define a dimensionless parameter

$$R = \frac{(Q_{\rm ecs} + F_{\rm rad})}{F_{\rm mech}}, \tag{19}$$

in which $F_{\rm rad}$ is the radiative energy flux incident on the interface. For SN1993J, the magnitude of R is $\sim 10^3$, it is dominated by heat conduction, based on the calculations above, and it is independent of time (subject to the continuing validity of the specific models used). For less steep SNRs (having smaller values of n), R decreases, but only a factor of about ten as one progresses from $n = 30$ to $n = 10$. As a result, the qualitative effect identified here seems likely to remain. In the high-flux experiment, $F_{\rm rad} \sim \sigma T_{\rm cs}^4$, as the electrons and ions are near equilibrium and the shocked layer is optically thick to the thermal radiation. When evaluated using Supplementary Equation 12, the conductive heat flux is a few times smaller than $F_{\rm rad}$. This was sufficient to produce the observed effects, discussed above.

In summary, it would appear that the energy fluxes are larger, in a dimensionless sense, in the emergent SNR than they are in the lab experiment. They have a noticeable effect in the lab experiment. We argue above that they seem likely to have a larger effect in the SNR.

Dimensionless Parameter	SN1993J at 0.1 yrs	NIF experiment
$\lambda_{\rm c}/L$	10^{-4}	10^{-8}
Reynolds number Re	$4 \times 10^5 t_{\rm yr}^{-0.48}$	10^7
Energy flux ratio R	10^3	2
Peclet number Pe	1	4
Ryutov number Ry	4	5

Supplementary Table 2: **Dimensionless parameters** Key parameters based on Supplementary Table 1.

Supplementary References

[1] Hogan, W. J., Moses, E. I., Warner, B. E., Sorem, M. S. & Soures, J. M. The National Ignition Facility. *Nuc. Fus.* **41**, 567–573 (2000).

[2] Dewald, E. L. *et al.* Dante soft x-ray power diagnostic for National Ignition Facility. *Rev. Sci. Instrum.* **75**, 3759–3761 (2004).

[3] Moore, A. S. *et al.* Developing high-temperature laser-driven half hohlraums for high-energy-density physics experiments at the National Ignition Facility. *Fus. Sci. Tech.* **63**, 76–81 (2013).

[4] Moore, A. S. *et al.* Radiation transport and energetics of laser-driven half-hohlraums at the National Ignition Facility. *Phys. Plasmas* **21** (2014).

[5] Doss, F. W. *et al.* The Shock/Shear platform for planar radiation-hydrodynamics experiments on the National Ignition Facility. *Phys. Plasmas* **22** (2015). 56th Annual Meeting of the APS Division of Plasma Physics, New Orleans, LA, OCT 27-31, 2014.

[6] Nagel, S. *et al.* A platform for studying the Rayleigh-Taylor and Richtmyer-Meshkov instabilities in a planar geometry at High Energy Density at the National Ignition Facility. *Phys. Plasmas* **24** (2017).

[7] Chevalier, R. A. Self-similar solutions for the interaction of stellar ejecta with an external medium. *Astrophys. J.* **258**, 790–797 (1982).

[8] Ensman, L. & Burrows, A. Shock breakout in SN1987A. *Astrophys. J.* **393**, 742–755 (1992).

[9] Tolstov, A., Blinnikov, S. I. & Nadyozhin, D. K. Coupling of matter and radiation at supernova shock breakout. *Mon. Not. R. Astronom. Soc.* **429**, 3181–3199 (2013).

[10] Chevalier, R. A. The interaction of the radiation from a Type II supernova with a circumstellar shell. *Astrophys. J.* **251**, 259–265 (1981).

[11] Suzuki, T. & Nomoto, K. X-rays from SN1993J and structures of ejecta and circumstellar medium. *Astrophys. J.* **455**, 658–669 (1995).

[12] Fransson, C., Lundqvist, P. & Chevalier, R. A. Circumstellar interaction in SN1993J. *Astrophys. J.* **461**, 993–1008 (1996).

[13] Chevalier, R. A. & Fransson, C. Emission from circumstellar interaction in normal type II supernovae. *Astrophys. J.* **420**, 268–285 (1994).

[14] Fransson, C. & Bjornsson, C. I. Radio emission and particle acceleration in SN1993J. *Astrophys. J.* **509**, 861–878 (1998).

[15] Baron, E., Hauschildt, P. & Branch, D. Modeling and interpretation of the optical and HST UV spectrum of SN1993J. *Astrophys. J.* **426**, 334–339 (1994).

[16] Bedogni, R. & Dercole, A. The electron thermal conduction in young supernova-remnants. *Astronom. Astrophys.* **190**, 320–332 (1988).

[17] Band, D. Self-similar supernova expansion with heat-transport. *Astrophys. J.* **332**, 842–856 (1988).

[18] Nymark, T. K., Fransson, C. & Kozma, C. X-ray emission from radiative shocks in type II supernovae. *Astronom. Astrophys.* **449**, 171–192 (2006).

[19] Drake, R. P. *High Energy Density Physics: Fundamentals, Inertial Fusion and Experimental Astrophysics* (Springer Verlag, 2006).

[20] Ryutov, D. D. *et al.* Similarity criteria for the laboratory simulation of supernova hydrodynamics. *Astrophys. J.* **518**, 821 (1999).

[21] Betti, R., Goncharov, V. N., McCrory, R. L. & Verdon, C. P. Growth rates of the ablative Rayleigh-Taylor instability in inertial confinement fusion. *Phys. Plasmas* **5**, 1446–1454 (1998).

[22] Bose, A., Woo, K. M., Nora, R. & Betti, R. Hydrodynamic scaling of the deceleration-phase Rayleigh-Taylor instability. *Phys. Plasmas* **22**, 072702 (2015).

[23] Modica, F., Plewa, T. & Zhiglo, A. The Braginskii model of the RayleigheTaylor instability. I. Effects of self-generated magnetic fields and thermal conduction in two dimensions. *High Energy Density Phys.* **9**, 767–780 (2013).

[24] Chevalier, R. A., Blondin, J. M. & Emmering, R. T. Hydrodynamic instabilities in supernova remnants: self-similar driven waves. *Astrophys. J.* **392**, 118–130 (1992).
